# Pelvic Exenteration in Advanced, Recurrent or Synchronous Cancers—Last Resort or Therapeutic Option?

**DOI:** 10.3390/diagnostics14161707

**Published:** 2024-08-06

**Authors:** Vlad Rotaru, Elena Chitoran, Daniela-Luminita Zob, Sinziana-Octavia Ionescu, Gelal Aisa, Prie Andra-Delia, Dragos Serban, Daniela-Cristina Stefan, Laurentiu Simion

**Affiliations:** 1 Medicine School, “Carol Davila” University of Medicine and Pharmacy, 050474 Bucharest, Romania; 2 General Surgery and Surgical Oncology Department I, Bucharest Institute of Oncology “Prof. Dr. Al. Trestioreanu”, 022328 Bucharest, Romania; 3 Medical Oncology Department I, Bucharest Institute of Oncology “Prof. Dr. Al. Trestioreanu”, 022328 Bucharest, Romania; 4 Surgery Department 4, Bucharest University Emergency Hospital, 050098 Bucharest, Romania

**Keywords:** pelvectomy, pelvic exenteration, radical pelvic surgery, survival, locally advanced pelvic cancer, cancer recurrence, long-term outcomes

## Abstract

First described some 80 years ago, pelvic exenteration remain controversial interventions with variable results and ever-changing indications. The previous studies are not homogenous and have different inclusion criteria (different populations and different disease characteristics) and methodologies (including evaluation of results), making it extremely difficult to properly assess the role of pelvic exenteration in cancer treatment. This study aims to describe the indications of pelvic exenterations, the main prognostic factors of oncologic results, and the possible complications of the intervention. **Methods:** For this purpose, we conducted a retrospective study of 132 patients who underwent various forms of pelvic exenterations in the Institute of Oncology “Prof. Dr. Al. Trestioreanu” in Bucharest, Romania, between 2013 and 2022, collecting sociodemographic data, characteristics of patients, information on the disease treated, data about the surgical procedure, complications, additional cancer treatments, and oncologic results. **Results:** The study cohort consists of gynecological, colorectal, and urinary bladder malignancies (one hundred twenty-seven patients) and five patients with complex fistulas between pelvic organs. An R0 resection was possible in 76.38% of cases, while on the rest, positive margins on resection specimens were observed. The early morbidity was 40.63% and the mortality was 2.72%. Long-term outcomes included an overall survival of 43.7 months and a median recurrence-free survival of 24.3 months. The most important determinants of OS are completeness of resection, the colorectal origin of tumor, and the presence/absence of lymphovascular invasion. **Conclusions:** Although still associated with high morbidity rates, pelvic exenterations can deliver important improvements in oncological outcomes in the long-term and should be considered on a case-by-case basis. A good selection of patients and an experienced surgical team can facilitate optimal risks/benefits.

## 1. Introduction

Pelvic exenteration is a very old surgical procedure (with a history of about 80 years—first reported in 1948 by Brunschwig [1]), with a controversial background, variable results, and ever-changing indications. Defined as the total or partial excision of the pelvic organs and regional lymphadenectomy, the procedure was initially used as a desperate attempt of treat locally advanced invasive pelvic malignancies but with very poor long-term outcomes and extreme mortality and morbidity rates [1,2]. After a better understanding of tumor biology was available and new advances were made in anesthesia and intensive care, transfusion and antibiotics, postoperative monitoring, and nutrition, pelvic exenterations became safer procedures and adequate options for radical treatment of such cases [3], and survival benefits were observed especially when negative resection margins could be achieved [4]. Ultra-radical compartmentalized resection became part of more specific and personalized surgical treatments [3,5,6,7,8], especially since most locally advanced or recurrent pelvic cancers have low sensitivity to chemoradiation. Given the fact that in these types of tumors only treated by chemoradiotherapy, death usually occurs in less than 2 years from diagnosis [9,10,11], pelvic exenterations became rational choices for treatment [5,12,13]. The progression of tumoral invasion usually respects embryological and anatomical borders that divide the pelvis into urological, gynecological, and digestive compartments, but once these boundaries are destroyed, the tumor will invade multiple anatomical spaces, thus resulting in the need for resecting all structures with the same embryological origin if a radical procedure is intended [14,15]. However, pelvic exenteration is still associated with significant rates of postoperative complications, but the results are quite difficult to interpret since the previous studies are not homogenous and have different inclusion criteria (different populations and different disease characteristics) and methodologies (including evaluation of results). Even the terminology used to describe various types of exenterations is not uniform, but there is currently a trend towards establishing a common terminology, which would ensure the reduction of heterogeneity between studies [16]. It is worth mentioning, however, the efforts of PelvEx Collaborative, which, for the first time, tried to clarify the role and outcomes of pelvic exenterations by analyzing large datasets obtained from multicenter collaboration across the globe and conducting systematic reviews of the existing literature [17,18,19,20,21]. In the era of personalized cancer care where tremendous importance is placed on the patient’s quality of life [22,23], concerns about the quality of life after this very invasive, mutilating surgical procedure [24,25] further cloud the discussion about the role it plays in locally advanced or recurrent pelvic cancers. This study aims to describe the indications of pelvic exenterations, the main prognostic factors of oncologic results, and the possible complications of the intervention, based on the “real-world” experiences of the Bucharest Institute of Oncology “Prof. Dr. Al. Trestioreanu”.

## 2. Materials and Methods

The Institutional Revied Board of Bucharest Institute of Oncology “Prof. Dr. Al. Trestioreanu”, Romania approved this study. For this study, all patients in our hospital who underwent pelvic exenteration between January 2013 and December 2022 were eligible for inclusion, regardless of indication (radical or palliative, malignant or benign disease, and primary advanced or recurrent pelvic cancer or synchronous pelvic cancers). Some patients were included despite having systemic dissemination of disease and pelvic exenteration was indicated as a palliative procedure with the purpose of ameliorating symptomatology. We excluded patients that required bone resection because in our institution we do not have a clinic of Orthopedy, and all such cases are addressed to other hospitals. Moreover, in the context of our limited experience with bone resections, there can be additional difficulties in analyzing data because of the added specific morbidity and mortality, which may lead to overestimation of peri-/postprocedural results and complications and to the underestimation of long-term oncological results. All included patients were over 18 years old and signed an informed consent for participation. A total of 132 patients were eligible for inclusion.

Data were retrieved by consulting the patient charts and records, the results of paraclinical imaging, and histopathological results. The surgery was ascertained from the procedure report. Data collected included sociodemographic information, comorbidities, pelvic exenteration indication, histopathological aspects, local and systemic dissemination of disease, curative/palliative intent of surgery, duration of surgery, blood-loss, duration of hospital stay, peri-/postoperative complications, mortality, the need for and type of neoadjuvant/adjuvant treatment, and long-term results after surgery like survival periods and time until recurrence/progression.

An assessment of patient characteristics, disease particularities, and peri-/postoperative details and complications was performed using data from all patients included. For the oncological outcomes analysis, only data from patients with histopathologically confirmed malignant disease were used.

The five benign cases (consisting of cancer patients who developed complexed fistulas between pelvic organs during/after cancer treatment but who did not have any sign of pelvic malignant involvement at the time of exenteration) were only included in the analysis of procedural details—such as length of operation, blood loss, post-operative complication or mortality. For oncological outcomes—such as overall survival and recurrence-free survival or radicality of procedure—we only included the 127 cases with known malignant involvement of pelvic organs at the time of exenteration.

### 2.1. Preoperative Management of Patients

All patients included were subjects of broad preoperative workups aimed at clarifying the diagnosis, evaluating the extent of the disease, and determining the feasibility of curative resection. In all patients with neoplastic disease, a histopathological confirmation of malignancy and an imaging evaluation of local and systemic dissemination were available prior to surgery. Each cancer case was discussed by a multidisciplinary tumor board and proper treatment plans were established on a case-to-case basis based on patient history, tumor characteristics, and tumoral load. Whenever appropriate, neoadjuvant treatment was proposed and administered to the patient. Surgery was performed after 4–8 weeks after the completion of neoadjuvant chemo/radiotherapy.

### 2.2. Surgery

**Figure 1:**
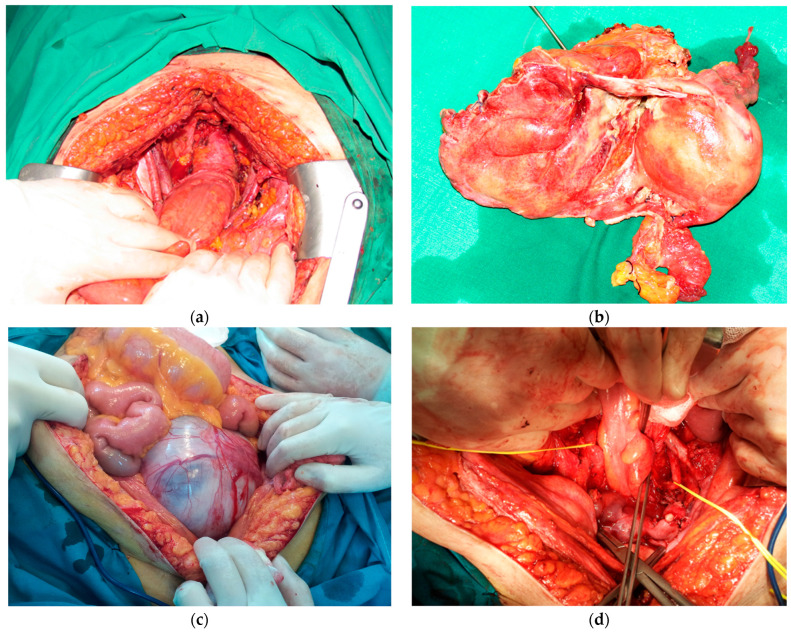
The role of the multidisciplinary surgical teams in pelvic exenterations: (**a**) Intraoperative aspect after an anterior pelvic exenteration for locally invasive cervical cancer (anterior invasion of urinary bladder) treated by neoadjuvant radiotherapy. (**b**) Same case as (**a**)—resection specimen. (**c**) Intraoperative aspect after median laparotomy in patient with synchronous malignant tumors of ovary and urinary bladder and heavy vascular pathology. (**d**) Same case as (**c**)—aspect after anterior pelvic exenteration; notice the presence of bilateral aorto-femoral Dacron prosthesis (the left one being placed in front of the left ureter, thus making surgical dissection more complex).

Patients underwent some form of pelvic exenteration under general anesthesia. All surgeries were performed by experienced oncologic surgeons skilled in both gynecologic and colorectal surgery. Whenever an anterior exenteration was performed, an experienced urologist was also co-opted. Vascular surgeons were called on each case in which vascular resection might have been needed or where vascular dissection was anticipated to be difficult. Pelvic exenterations often involve multidisciplinary teams for adequate management of the patient and optimization of the results (Figure 1).

Total exenteration was defined as the removal of all pelvic organs with or without pelvic floor resection. Partial resections were defined as the removal of internal genital and urinary organs (anterior pelvectomy) or as the removal of internal genital organs together with colorectal resection (posterior pelvectomy). The organs were removed “en-bloc” together with the pelvic tumor. A curative resection (R0 resection) was defined as surgery resulting in a histopathological specimen microscopically proven to have tumor-free margins greater than 1 mm. R1 (malignant cells evident on microscopic examination of resection margins) and R2 (macroscopic residual tumor) were considered palliative resections. All efforts were made to ensure R0 resection, including resection of neighboring organs affected by the disease and resection of the pelvic floor or regional lymphadenectomies, as needed.

### 2.3. Postoperative Follow-Up

Perioperative morbidity and mortality were defined as events occurring within 30 days from surgery. Late morbidity was defined as all adverse effects after 30 days. Adverse events were classified according to the Clavien–Dindo classification [26]. Grades 1–2 were considered minor complications, while Grades 3–5 were defined as major complications. Regular follow-ups were scheduled for all patients every 3–6 months consisting of clinical examination, imaging and endoscopic evaluation, and biochemical analysis. Follow-up continued until death or until the patient did not return for further medical visits. The length of follow-up varied from 12 to 120 months.

Additional survival data were obtained by contacting the patient or their family contacts and by inquiries made with the Public Service for Records of Persons.

Adjuvant therapies were indicated and administered as needed for each patient after discussing the case with the multidisciplinary tumor board.

### 2.4. Statistical Analysis

Categorical data were compared using the Chi square test, and their distribution was presented as absolute and relative frequencies. Continuous data were analyzed as means and standard deviations. Oncological results like overall survival (OS) and the recurrence-free survival rate (RFS) were analyzed and a comparison between various subsets of patients was performed. Overall survival was defined as the time between surgery and death. RFS was defined as the time between surgery and the date of diagnosis of local or distant disease recurrence. Patients were censored at the time of known death or time at which the patient was lost from follow-up. Factors potentially influencing oncologic and postoperative results were analyzed by Cox proportional hazard regression models. Factors found to be significantly linked in univariant analysis were further analyzed by multifactorial regression models. A *p*-value lower than 0.05 was considered to be the threshold for statistical significance.

## 3. Results

Among the 132 patients included in this study, the median age at the time of pelvic exenteration was 57.4 years (with extremes between 24 and 78 years. Most patients (51.52%) were older than 60 years. The gender distribution of patients was F/M = 1.93/1. A quarter of all patients lived in urban areas—this aspect is expected in rural areas as access to healthcare services is known to be lower, resulting in delayed diagnostic and more advanced stages at the time of diagnostic. Given the more advanced age of some of the patients included, some comorbidities were a factor, the most prevalent being arterial hypertension (found in almost half of the subjects). Other comorbidities encountered were diabetes (controlled either by oral antidiabetic medication or by insulin subcutaneous injections), chronic renal disease (usually associated with urinary blockage by ureteral compression by the pelvic tumor and secondary hydronephrosis), and chronic pulmonary disease (in relation with smoking habits and exposure to toxic atmospheric/occupational agents). The sociodemographic characteristics of the patients are summarized in Table 1.

Pelvic exenteration was indicated in most cases (127) for malignant disease—65.15% of those with primary cancers, 28.03% with recurrent cancer, and 3.03% with multiple synchronous primary cancers of pelvic organs. Five patients had a pelvic exenteration performed for benign indications (complex fistulas between various pelvic organs). It is worth mentioning that the five patients with complex fistulas between various pelvic organs are also patients who had prior therapy for cancer (including surgery or chemoradiotherapy)—but in their cases, there was no evidence of recurrence of pelvic disease at the time of exenteration. In these cases, the length of time between the initial treatment and formation of the complex fistular complication varied between 3 and 14 months. The origin of the neoplastic disease was most often a locally advanced or recurrent gynecological malignancy (53.95%: forty-five cases—cervical, twelve cases—endometrial, nine cases—ovarian, and two cases—vaginal). Pelvic exenteration was also indicated in thirty-six cases of colorectal cancer (28.35%), twenty-one cases of malignant tumor of the urinary bladder (16.53%), and two cases of soft tissue sarcomas (1.98%). The high proportion of gynecological cancer in our cohort can be explained by the addressability of cancer cases to our institute (one-third of all surgical procedures are performed for gynecological malignancies). The most common histological type was adenocarcinoma (around 45% of all cases), followed by squamous cell carcinoma. The differentiation degree of tumors was G3 in almost half of cases. Only 10% of tumors were well differentiated. This aspect was to be expected given the correlation between poorly differentiated tumors and highly invasive forms of disease. All patients included were discussed with a multidisciplinary tumor board and adequate neoadjuvant therapies were proposed and administered to the patients on a case-by-case basis. As a result, 56.06% of patients (74) received neoadjuvant therapy consisting of chemotherapy, radiotherapy, brachytherapy, or a combination of the above. The indications for which pelvic exenterations were performed and the characteristics of the neoplastic disease are summarized in Table 2.

As for the type of exenteration performed, thirty-eight patients had a total pelvic exenteration and ninety-four underwent a partial pelvectomy. In over 52% of cases (sixty-nine patients) an anterior pelvectomy was needed, almost 29% needed a total pelvectomy (thirty-eight cases in total; in seven of these cases, pelvic floor resection was also required, while in thirty-one cases, the resection was performed in a supra-levatorian plane), and the rest underwent posterior exenteration (twenty-five patients, representing almost 19%). In two cases, lateral extension of the resection was needed (segmental resection of external iliac artery and/or vein with vascular reconstruction). In 92 patients (72.44%), a regional lymphadenectomy was performed—pelvic bilateral, para-aortic, inguinal bilateral, or a combination of the above-mentioned. Regional lymphadenectomy was not needed in 35 patients. Resection of additional extra-pelvic organs was required in 16.67% of cases and this involved segmental ileal resection (twelve cases), appendectomy (two cases), colectomy (two cases), vulvectomy (one case), greater omentum (four cases), and splenectomy (one case). All efforts were made to ensure radical resection whenever feasible. As a result, most cases (76.38%–97 cases) were in fact radical procedures and only 30 patients had a palliative procedure (in 17 cases the palliative character of the exenteration was known prior to surgery and in 13 cases the histopathological result showed a R1 resection type, thus proving the palliative nature of the procedure). In three cases, residual macroscopic tissue was present (R2 resection). Restoration of the digestive tract was performed in 63 cases either by colorectal anastomosis (option of choice whenever possible) or by terminal colostomy (when suture was not possible due to deficient vascularization of anastomotic partners, poor-quality tissues due to post-radiation effects, and patient-related factors like extreme obesity or protein malnourishment). Colorectal anastomosis was performed manually in sixteen cases and mechanically in in six cases. Urinary reconstruction was performed in 107 cases—“gun barrel” bilateral cutaneous ureterostomy (39.25%), Bricker ileal conduit (41.12%), and ileal neobladder (19.63%).

There was no significant difference in the duration of the procedure between radical and palliative procedures nor between exenterations performed for primary and recurrent cancers (*p*-value = 0.342 and 0.566, respectively). The medium length of surgery was 410 min (range 280–600 min). The blood loss was higher in cases with recurrent disease when compared to primary cancer (*p*-value = 0.05). The median estimated blood loss during surgery was 850 mL (range 350–1400 mL). The median stay in the intensive care unit was 7 days (range 3–32 days) and the median in-hospital stay was 17 days (range 10–54 days). Longer admissions into the intensive care units were associated with reinterventions (*p*-value < 0.001), septic complications (*p*-value = 0.02) and organ failure (*p*-value < 0.001).

Early morbidity and mortality were defined as events occurring within 30 days from surgery, and their severity was assessed according to the Clavien–Dindo classification where Grades 1–2 mean a minor complication; Grades 3–4 mean a major, life-threatening complication; and Grade 5 means death. Minor adverse effects occurred in 25 patients (18.94%) and major complications occurred in 29 patients (21.69%). The complications can be classified into medical (deep vein thrombosis, prolonged post-op ileus, acute cardiac and pulmonary complications, organ failure, pseudomembranous colitis, and allergic reaction to medication) and surgical (post-op hemorrhage, digestive and urinary fistular complications, septic complications—peritonitis and intraabdominal abscess, evisceration, and acute lower limb ischemia). Early mortality (within 30 days from procedure) occurred in three cases (2.72%) because of septic complications followed by multiple system organ failure or acute cardiac events. Intraoperative aspects, early morbidity, and mortality outcomes after pelvic exenterations are summarized in Table 3. The 90-day mortality rate was 1.52% (two cases), with death occurring because of thromboembolic complications or septic complications.

All resected specimens were analyzed, and histopathologic reports were provided. The size of tumors was higher in recurrent cases when compared to primary tumors (*p*-value = 0.05). The radical nature of the exenteration was confirmed by microscopy in 97 cases and disproved in 30 cases (in 13 of these, the surgery had curative intent, but the resection specimen showed microscopical malignant foci). Radicality was more likely to be achieved when the lateral extension of the disease (invasion of vascular structures or bone and extensive involvement of parametrial or pararectal structures) was absent (*p*-value = 0.05). The results of histopathological analysis of resection specimens are summarized in Table 4.

After surgery, all cases were rediscussed with a multidisciplinary tumor board, and for 93 patients, it was deemed necessary to provide adjuvant therapy (chemotherapy, radiotherapy, immunotherapy, or a combination of the above). There was no significant difference in the need for adjuvant therapy between primary and recurrent tumors (*p*-value = 0.213). The median post-op follow-up was 32 months (range 6–120 months). Follow-up ended either by the death of the patient or when the patient was lost from follow-up. Local recurrences were observed in 37 patients after a median period of 15 months. Among those, as expected, 30 were patients had known remanent tumoral tissue after the exenteration (R1/R2 resection). The local recurrence rate is higher in patients with positive resection margins (*p*-value < 0.001) and in patients operated on for recurrent malignant disease (*p*-value = 0.03). Systemic recurrence was observed in 21 patients after a median period of 23 months. Among these, 13 patients had systemic disease at the time of palliative pelvic exenteration performed for the control of symptoms.

The analysis of long-term oncological outcomes showed a median OS (overall survival) of 43.7 months and a median RFS (recurrence-free survival) of 24.3 months. Among our cohort we have experienced 2 patients which by all intent and purpose were cured, them being alive at 120 months after surgery.The most important determinants of OS in the univariate analysis were R0 resection (52.6 months versus 14.8 months in patients with R1/R2 resection, *p*-value < 0.001), the primary origin of the tumor (48.6 months in colorectal cancers versus 38.3 months in gynecological cancers versus 35.2 months in urinary bladder cancers), and the primary characteristics of the tumor (49.1 months versus 32.8 months in recurrent cancers, *p*-value = 0.04). The dimension of tumor, histopathological type, and the degree of differentiation and perineural invasion did not significantly affect OS. Lymphovascular invasion on resected specimens was associated with decreased OS (*p*-value = 0.02).

Late-onset complications (>30 days) appeared in our group. Most consisted in malfunctioning of stomas and repetitive urinary infections. A total of 14 patients required late interventions due to bowel obstruction (due to benign causes like fibrotic changes in their bowel with subsequent stenosis, adhesions, the fixation of the small intestine in the pelvic cavity with angulation of the bowel, or malignant causes such as carcinomatosis or pelvic recurrence). Another 21 patients required intervention due to eventration (median or parastomal), septic complications (pelvic/ischiorectal abscesses and peritonitis), fistular complications, or recurrence of disease. 

Subgroup analysis showed significant differences between the various types of tumors treated and types of exenterations. These differences are summarized in Table 5.

## 4. Discussion

Our analysis of this procedure showed that even though, over the years, significant improvements have been made to both surgical technique and postoperative patient management, pelvic exenteration remains associated with increased morbidity. In our study, the overall morbidity rate was 40.63%, and the complications can be classified into medical (deep vein thrombosis, prolonged post-op ileus, acute cardiac and pulmonary complications, organ failure, pseudomembranous colitis, and allergic reaction to medication) and surgical (post-op hemorrhage, digestive and urinary fistular complications, septic complications—peritonitis and intraabdominal abscess, evisceration, and acute lower limb ischemia). Seventeen patients required reinterventions for expected causes like occlusion, digestive/urinary fistulas, hemostasis, and peritonitis/intraabdominal abscess and, in some cases, more patient-specific complications like acute ischemia of inferior limb (in patient with history of multiple vascular interventions). The morbidity and reintervention rates are smaller than in other studies, highlighting the importance of performing exenterative surgery in specialized centers with highly experienced oncological surgeons [27]. Early mortality occurred in three cases (2.72%) because of septic complications followed by multiple system organ failure; acute cardiac events are also within the limits presented in the recent literature.

Our study confirms the long-term benefits pelvic exenterations can induce in patients with locally advanced, recurrent, or synchronous pelvic cancers. In situations where clinicians are faced with a paucity of therapeutic solutions, pelvic exenteration can provide overall and recurrence-free survival benefits, and clear resection margins are the most important determinant of these results [28,29,30,31]. Advances in vascular and orthopedic surgery have allowed for more extensive resection in order to achieve R0 resection [32,33,34,35]. When bone resections are employed, these influence both surgical results and oncologic outcomes. But, there are some previous studies suggesting that an R0 resection is more likely in colorectal malignancies [36] and in non-recurrent settings [28,29,37]. The majority of studies prove superior survival rates for primary cancers when compared to recurrent tumors (estimated 5-year survival rate of 43–77% for primary and 8–41% for recurrent lesions) [38,39,40]. Similar results have been observed in our study too. It has been widely proven that negative resection margins play a crucial role in long-term outcomes [4,41], and every effort should be made to achieve it including “en-bloc” resection of extra pelvic organs or of vascular/bone structures. In some cases with heavy pre-irradiation, achieving R0 may be challenging due to difficulties in distinguishing between the tumoral tissue, the post-radiation fibrosis, and inflammation. Studies have described additional factors that influence long-term outcomes, like tumoral volume or dimension, but in this aspect, the published results are inhomogeneous and conflicting. While some studies do not consider the size of the pelvic tumor as a determinant of overall survival rates [42], others consider that tumoral size plays a role [5]. Other factors include the histological subtype of the tumor [43] and various pathological findings. In our study, the dimensions of the tumor, the histopathological type, and the degree of differentiation and perineural invasion did not significantly affect OS. Lymphovascular invasion on resected specimens was associated with decreased OS, similar to other previous studies.

As expected, the long-term follow-up in our study demonstrated local recurrences are more likely in patients with known tumoral tissue after the exenteration (R1/R2 resection). The local recurrence rate is higher in patients with positive resection margins and in patients operated on for recurrent malignant disease. Systemic recurrence was observed after a median period of 23 months, but our results can be influenced by the fact that we have included in our cohort patients with known metastatic disease in which pelvic exenteration was performed for symptom control. A higher disease-free survival is usually associated with primary disease, and tumors of colorectal origin have better survival rates when compared to other recurrent or primary tumors [41,44], with results comparable to ours.

### Strengths, Limitations and Future Directions of Study

The strength of this study comes from having a large study group, allowing for a better capacity for statistical analysis. Moreover, all patients were selected from a single tertiary center and were operated on by a limited number of highly trained oncological surgeons, thus limiting the potential influence of a “learning curve”, which can influence R0 resection rates.

A limitation of our study is the retrospective character and the high heterogeneity of included patients, which predispose type 2 statistical errors. However, given the fact that pelvic exenterations are not so common, we decided to include both radical and palliative procedures, various tumoral forms and histological aspects, and all types of exenteration in our study. Another limitation of our study is the exclusion of cases that required bone resections due to limited specialized personnel—this type of patients is an important subgroup, and their inclusion/exclusion can in fact modify the overall results of the procedure and the oncological outcomes.

Future directions aimed at reducing heterogeneity would involve transitioning from retrospective to prospective study design (currently, in the Bucharest Institute of Oncology, we are conducting a prospective study on the role and outcomes of pelvic exenterations, which started in 2022, involving a more standardized data collection system, data about quality of life after the procedure, and an extensive follow-up period (10 years) to capture long-term outcomes and late-onset complications). In this study, design control groups were introduced, which included all patients for whom a pelvic exenteration was be recommended but the patient decided to forgo in the absence of any medical contraindication for the procedure.

## 5. Conclusions

Exenterations, although rational procedures for locally advanced, recurrent, or synchronous pelvic malignancies in the absence of other efficient therapeutic options, remain associated with high morbidity and poor post-procedural quality of life. However, a significant survival benefit is observed when an R0 resection is possible, the primary tumor is colorectal in origin, the tumor is primary rather than recurrent, and there is no lateral extension or lymphovascular invasion. Given the observed survival benefits and the possibility of selecting the patients, we consider pelvic exenterations to be safe and feasible procedures in locally advanced, recurrent, and synchronous pelvic malignancies; they should be considered on a case-to-case basis.

## Figures and Tables

**Table 1:** Sociodemographic characteristics of patients.

**Age**	<40 years	11 (8.33%)
57.4 years (range 24–78)	40–49 years	24 (18.18%)
	50–59 years	29 (21.97%)
	60–69 years	35 (26.52%)
	≥70 years	33 (25.00%)
**Gender**	Female	87 (65.91%)
	Male	45 (34.09%)
**Provenience**	Urban	34 (25.76%)
	Rural	98 (74.24%)
**Comorbidities**	Hypertension	48 (36.36%)
	Diabetes	19 (14.39%)
	Chronic renal disease	12 (9.09%)
	Chronic pulmonary disease	9 (6.82%)

**Table 2:** Exenteration indication and neoplastic disease characteristics.

**Indication for exenteration**	Primary neoplasm	86 (65.15%)
	Recurrent cancer	37 (28.03%)
	Synchronous primary cancers	4 (3.03%)
	Benign indications *	5 (3.79%)
**Origin of tumor**	Cervical	45 (35.43%)
	Endometrial	12 (9.45%)
	Ovarian	9 (7.09%)
	Urinary bladder	21 (16.53%)
	Colorectal (including anal)	36 (28.35%)
	Other **	4 (3.15%)
**Histological type**	Squamous cell carcinoma	48 (36.36%)
	Adenocarcinoma	59 (44.70%)
	Sarcoma	4 (3.03%)
	Urothelial carcinoma	21 (15.91%)
**Differentiation degree**	G1—well differentiated	14 (10.61%)
	G2—moderately differentiated	56 (42.42%)
	G3—poorly differentiated	62 (46.97%)
**Neoadjuvant therapy**	Yes	74 (56.06%)
	No	58 (43.94%)

* Includes complex fistulas between various pelvic organs in previously treated cancer patients with no evidence of pelvic tumor at the time of exenteration; ** all other tumoral types including vaginal cancers or soft tissue sarcomas.

**Table 3:** Intraoperative aspects and early morbidity and mortality outcomes after pelvic exenterations.

**Type of Pelvic Exenteration**	
Total exenterations	38 (28.79%)
Total without pelvic floor resection	31 (23.49%)
Total infralevator	7 (5.30%)
Partial exenterations	94 (71.21%)
Anterior	69 (52.27%)
Posterior	25 (18.94%)
**Curative Intent**	
Radical	97 (76.38%)
Palliative	30 (23.62%)
**Lymph node dissection**	
Pelvic lymph node	85 (66.93%)
Paraaortic lymph node	21 (16.54%)
Inguinal lymph node	2 (1.57%)
**Resection of extra pelvic organs**	
Yes	22 (16.67%)
No	110 (83.33%)
**Digestive tract reconstructive procedures**	63
Colorectal anastomosis	19 (30.16%)
Terminal colostomy	44 (69.84%)
**Urinary reconstructive procedures**	107
Cutaneous urostomy	42 (39.25%)
Bricker procedure	44 (41.12%)
Ileal neobladder	21 (19.63%)
**Median length of surgery (min)**	410 min (range 280–600 min)
**Median blood loss (mL)**	850 mL (range 350–1400 mL)
**Median intensive care unit stay (days)**	7 days (range 3–32 days)
**Median stay in hospital (days)**	17 days (range 10–54 days)
**Early morbidity and mortality**	
Clavien–Dindo Grades 1–2	25 (18.94%)
Clavien–Dindo Grades 3–4	29 (21.69%)
Reinterventions	17 (12.88%)
Causes of early reinterventions	occlusion, digestive/urinary fistulas, hemorrhagic shock, peritonitis/intraabdominal abscess, and acute ischemia of inferior limb
Mortality (Clavien-Dindo Grade 5)	3 (2.72%)
Causes of early mortality	multiple system organ failure, acute cardiac complications

**Table 4:** Histopathological results for resected specimens in cancer cases.

**Tumor size**	<5 cm	38 (29.92%)
	≥5 cm	89 (70.08%)
**Radicality**	R0	97 (76.38%)
	R1	27 (21.26%)
	R2	3 (2.36%)
**Lateral extension of tumor**	Yes	23 (18.11%)
	No	104 (81.89%)
**Positive regional lymph nodes**	Yes	95 (74.80%)
	No	32 (25.20%)
**Perineural invasion**	Yes	69 (54.33%)
	No	36 (28.35%)
	Not specified	22 (17.32%)
**Lympho-vascular invasion**	Yes	78 (61.42%)
	No	42 (33.07%)
	Not specified	7 (5.51%)

**Table 5:** Subgroup analysis of pelvic exenterations outcomes in cancer cases.

**By Type of Exenteration**		
**Total vs. partial** 38 vs. 94 cases	Early-morbidity rate	39.47% vs. 41.49% (*p* = 0.048)
	Early-mortality rate	2.63% vs. 2.13% (*p* = 0.238)
	RFS	23.9 months vs. 23.0 months (*p* = 0.146)
	OS	40.3 months vs. 42.7 months (*p* = 0.047)
**Radical vs. palliative** (R0 vs. R1/R2 resections) 97 vs. 30 cases	Early-morbidity rate	41.24% vs. 46.67% (*p* = 0.041)
	Early-mortality rate	2.06% vs. 3.33% (*p* = 0.034)
	RFS	31.8 months vs. 0 months (*p* < 0.001)
	OS	52.6 months vs. 14.8 months (*p*-value < 0.001)
**By Type of Tumor**		
**Primary vs. recurrent**	Early-morbidity rate	35.56% vs. 62.86% (*p* < 0.001)
90 vs. 37 cases	Early-mortality rate	1.11% vs. 5.40% (*p* < 0.001)
	RFS	26.6 months vs. 18.7 months (*p* = 0.03)
	OS	49.1 months vs. 32.8 months (*p*-value = 0.040)
**Colorectal vs. non-colorectal**	Early-morbidity rate	36.11% vs. 45.05% (*p* < 0.001)
36 vs. 91 cases	Early-mortality rate	2.78% vs. 2.19% (*p* = 0.187)
	RFS	28.2 months vs. 22.7 months (*p* = 0.024)
	OS	48.6 months vs. 38.3 months (in gynecological cancers—*p* < 0.001) or 35.2 months (in urinary cancers—*p* < 0.001)

Abbreviations used in table: R0 resection—no microscopic/macroscopic residual tumor after surgical resection (indicating the radical character of procedure); R1 resection—microscopic residual tumors after surgery; R2 resection—macroscopic residual tumor after surgery; RFS—recurrence-free survival; OS—overall survival.

## Data Availability

The raw data supporting the conclusions of this article will be made available by the authors on request.
